# Modeling Parkinson's disease‐related symptoms in alpha‐synuclein overexpressing mice

**DOI:** 10.1002/brb3.2628

**Published:** 2022-06-01

**Authors:** Agata Aniszewska, Joakim Bergström, Martin Ingelsson, Sara Ekmark‐Lewén

**Affiliations:** ^1^ Department of Public Health and Caring Sciences Molecular Geriatrics Uppsala University Uppsala Sweden; ^2^ Krembil Brain Institute University Health Network Toronto Ontario Canada; ^3^ Department of Medicine and Tanz Centre for Research in Neurodegenerative Diseases University of Toronto Ontario Canada

**Keywords:** alpha‐synuclein, behavioral test, ethology, Parkinson´s disease, transgenic mouse models, translational research

## Abstract

**Background:**

Intracellular deposition of alpha‐synuclein (α‐syn) as Lewy bodies and Lewy neurites is a central event in the pathogenesis of Parkinson's disease (PD) and other α‐synucleinopathies. Transgenic mouse models overexpressing human α‐syn, are useful research tools in preclinical studies of pathogenetic mechanisms. Such mice develop α‐syn inclusions as well as neurodegeneration with a topographical distribution that varies depending on the choice of promoter and which form of α‐syn that is overexpressed. Moreover, they display motor symptoms and cognitive disturbances that to some extent resemble the human conditions.

**Purpose:**

One of the main motives for assessing behavior in these mouse models is to evaluate the potential of new treatment strategies, including their impact on motor and cognitive symptoms. However, due to a high within‐group variability with respect to such features, the behavioral studies need to be applied with caution. In this review, we discuss how to make appropriate choices in the experimental design and which tests that are most suitable for the evaluation of PD‐related symptoms in such studies.

**Methods:**

We have evaluated published results on two selected transgenic mouse models overexpressing wild type (L61) and mutated (A30P) α‐syn in the context of their validity and utility for different types of behavioral studies.

**Conclusions:**

By applying appropriate behavioral tests, α‐syn transgenic mouse models provide an appropriate experimental platform for studies of symptoms related to PD and other α‐synucleinopathies.

## Graphical abstract

This review describes behavioral tests and procedures used to assess the most common motor and nonmotor symptoms observed in alpha‐synuclein transgenic mouse models of Parkinson's disease.

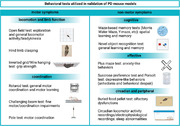

## 1 INTRODUCTION

Alpha‐synucleinopathies, including Parkinson´s disease (PD), PD with dementia (PDD) and dementia with Lewy bodies (DLB), are characterized by a progressive accumulation of alpha‐synuclein (α‐syn) as intraneuronal inclusions in different brain areas. Parkinson's disease is the most common α‐synucleinopathy and was originally described in the beginning of the 19th century by the British surgeon James Parkinson as “the shaking palsy” (Parkinson, 2002). As one of the key features of the PD brain, dopaminergic neurons in substantia nigra pars compacta (SNpc) are lost, which leads to bradykinesia, resting tremors, postural instability, muscular rigidity, and gait disturbances (Kalia & Lang, 2015). In addition, PD patients often develop various degrees of cognitive symptoms when the pathology has spread to neocortical areas. Apart from leading to great suffering for the patients, PD is causing a heavy burden on society, with currently approximately 10 million people affected worldwide (reviewed in [Balestrino & Schapira, 2020]). As of yet, there is no treatment against the underlying disease processes, although novel molecular insights have led to development of new drug candidates that are currently under evaluation in clinical trials.

## 2 THE CENTRAL ROLE OF α‐SYN AGGREGATION

**FIGURE 1:**
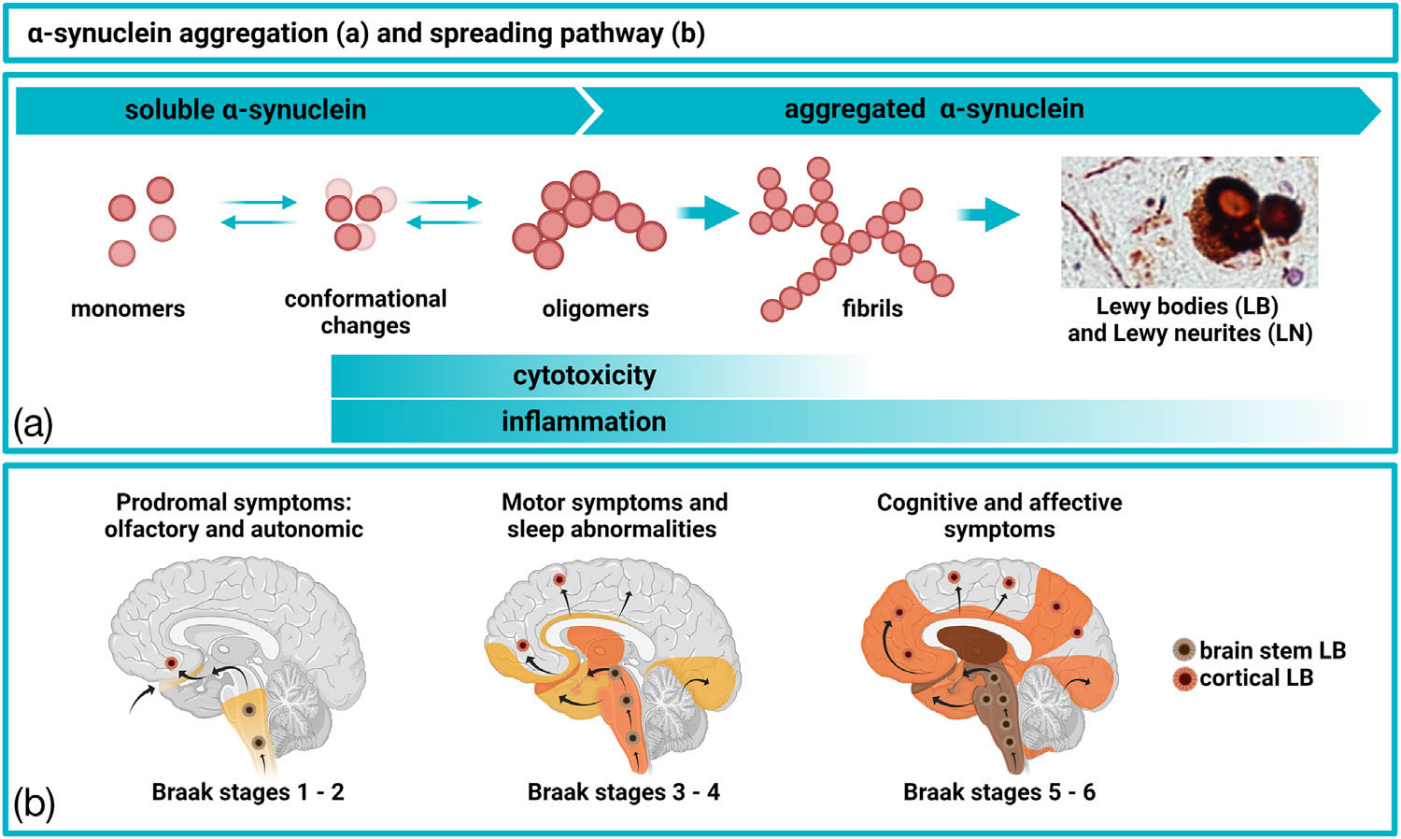
Aggregation pathway, toxicity (a) and propagation (b) of α‐synuclein in the PD brain. Created with BioRender

The identification of α‐syn as the main component of Lewy bodies and Lewy neurites (Figure 1A) was made about 25 years ago (Spillantini et al., 1997). In PD, these rounded inclusions between 5 and 25 μm are mainly located in the upper part of the brainstem, but can also be found in neocortex at later disease stages (reviewed in [Jellinger, 2018]). Genetic factors can affect the risk for PD and mutations in several genes, such as *SNCA* (the gene encoding α‐syn), *LRRK2*, *Parkin* and *PINK 1*, causing either dominant or recessive forms of PD (Moore et al., 2005), while multiplications of *SNCA* cause either PD or DLB (reviewed in [Rosborough et al., 2017]). Importantly, most of the genetic causes result in α‐syn pathology, pointing to a central role of this protein in the pathogenesis of PD and other α‐synucleinopathies (reviewed in [Houlden & Singleton, 2012]).

While the exact molecular mechanisms involved are incompletely understood, presynaptic aggregation of α‐syn has been suggested to have a direct neurotoxic effect in the affected brain (Colom‐Cadena et al., 2017; Kramer & Schulz‐Schaeffer, 2007). Pre‐fibrillar oligomeric forms of α‐syn (Figure 1A) seem to be particularly damaging and were shown by us and others to have toxic effects on cells and tissues ex vivo (Diogenes et al., 2012; Nasstrom et al., 2011). Such forms of α‐syn may also spread between cells and thereby propagate α‐syn pathology to interconnected brain areas (Figure 1B) (Braak et al., 2003). By such as‐of‐yet not fully understood mechanisms, α‐syn pathology can progress from the lower brainstem toward the medulla, midbrain, and basal forebrain before reaching the cerebral cortex (Figure 1B) (McCann et al., 2016).

Despite the fact that α‐syn is expressed throughout the brain, dopaminergic neurons seem particularly vulnerable to α‐syn‐related pathology, which indicates that α‐syn is involved in the regulation of dopamine metabolism. It has also been suggested that presynaptic α‐syn may affect the homeostasis of dopaminergic neurons by regulating neurotransmitter release via the SNARE complex and the trafficking of synaptic vesicles (Burre et al., 2010; Goedert et al., 2013; Uchihara & Giasson, 2016). In addition, data suggest that dysregulation of dopamine may contribute to the formation of α‐syn fibrils (Bridi & Hirth, 2018) and oligomers (Mor et al., 2017). Thus, evidence suggests a bidirectional interaction between α‐syn accumulation and dopamine dysfunction, leading to the synaptic dysregulation and progressive neurodegeneration observed in PD patients (Ingelsson, 2016). However, the exact nature of the molecular interaction between α‐syn and dopamine in the PD pathogenesis still remains unknown.

The central role of α‐syn in the neurodegenerative process has led to development of novel treatment strategies, aimed at reducing α‐syn pathology and limiting its propagation in the CNS. One of the treatment strategies is represented by passive immunotherapy. Aside from aiding in microglia‐mediated extracellular clearance of α‐syn in vitro and in vivo, treatment with an antibody against the C‐terminus of α‐syn was found to prevent cell‐to‐cell transmission of α‐syn (Bae et al., 2012) and to ameliorate motor symptoms (Games et al., 2014) in transgenic (tg) mice. Furthermore, in collaboration with BioArctic AB, we developed oligomer/protofibril selective α‐syn antibodies (Fagerqvist et al., 2013) and found that treatment with such antibodies can reduce levels of aggregated α‐syn in a cell model (Nasstrom et al., 2011) as well as in the central nervous system (CNS) of an *SNCA* tg mouse model (Lindstrom et al., 2014; Nordstrom et al., 2021).

**FIGURE 2:**
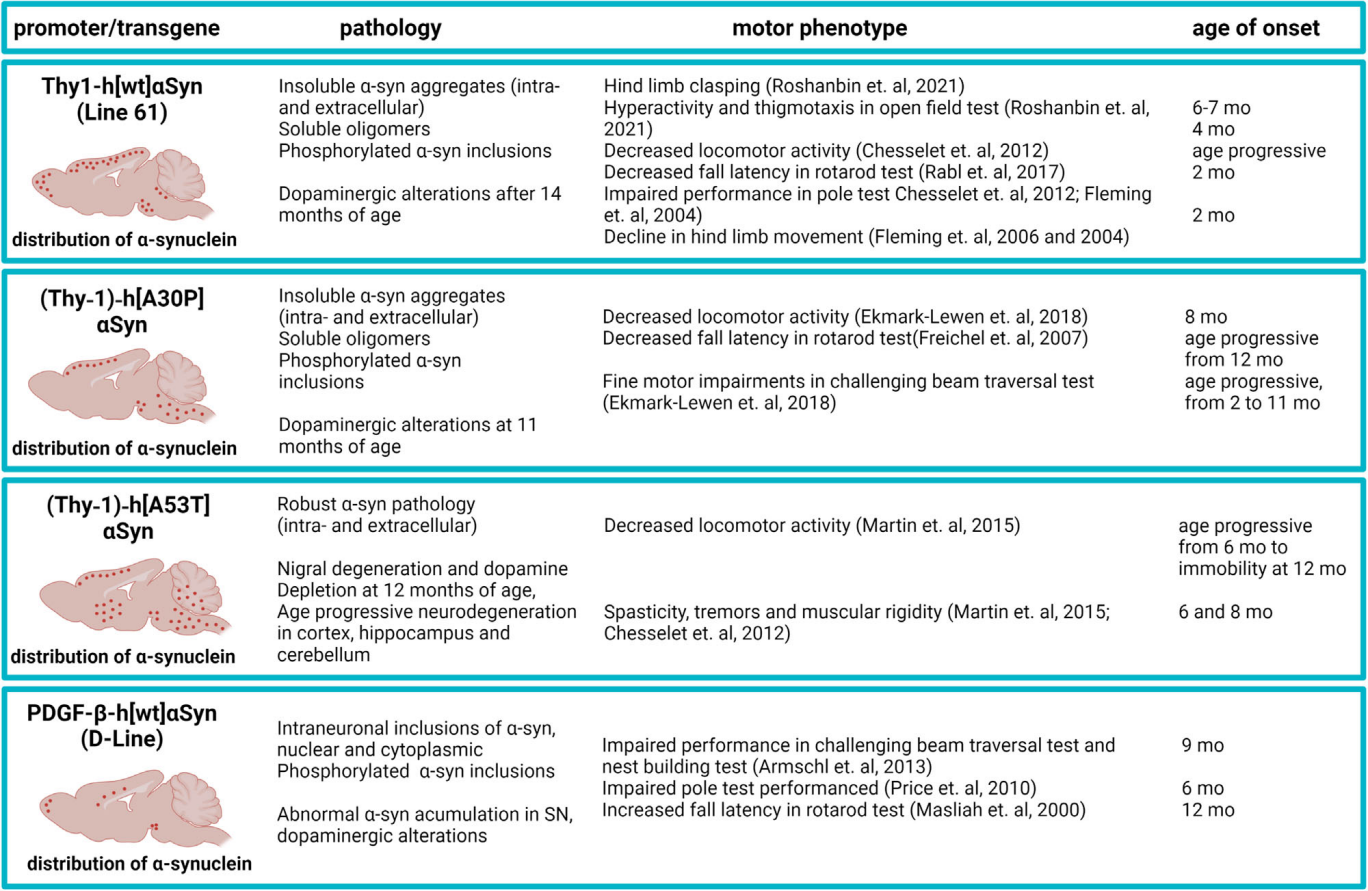
Brief characterization of selected SNCA transgenic mouse models of PD, including the pattern of α‐synuclein pathology, motor symptoms, and age of symptom onset. Created with BioRender

Various mouse models are extensively used in laboratories to gain understanding of α‐syn pathology and its relation to symptoms (Figure 2). Behavioral testing in mice is dependent on many different factors that can affect the results and lead to large within‐group variations. To facilitate the process of choosing suitable tests and make an adequate study design, the present review will focus on how symptoms are being assessed in α‐syn overexpressing mice. We will reflect upon what we can and cannot model in mice, with respect to both traditional and modern behavioral tests. Further, we will discuss ethoexperimental and multivariate approaches and how to measure different aspects of evolutionarily conserved strategies for survival in rodents as well as their relevance to preclinical study design.

## 3 TRANSGENIC MOUSE MODELS OVEREXPRESSING α‐SYN

Despite the fact that most PD cases are of sporadic origin, transgenic mouse models have proven to be a useful tool in studying basic mechanisms of PD‐related neuropathology. Since many different genes are associated to PD, several models have been developed. Overexpressing human wild‐type (WT) and different *SNCA* mutations in mice have high construct validity for studies of α‐syn‐induced neurodegeneration (reviewed in [Chesselet & Richter, 2011]). Most of these models develop relevant pathology consisting of aggregated α‐syn inclusions that bear resemblance to those in the affected human brain, but mostly with no obvious dopaminergic cell loss. Only mice expressing the artificial oligomerization‐prone mutants *E35K* and *E57K* display extensive dopaminergic cell loss in mesencephalon, whereas other mice expressing either human WT or the pathogenic A30P, A53T, or E46K mutants do not display any dopaminergic cell loss (Winner et al., 2011). Some of the most commonly used tg mouse models of PD are characterized in Figure 2.

Various tg mouse models of α‐syn overexpression are currently available, each with its own advantages and disadvantages in terms of the nature of α‐syn pathology, dopamine alterations, and behavioral abnormalities. In our laboratory, we are breeding two different mouse lines: the Thy1‐αSyn (L61) line, overexpressing WT human SNCA (Masliah et al., 2000) and the (Thy‐1)‐h[A30P] α‐syn (A30P) tg line expressing *SNCA* with the A30P mutation causing familial PD (Kahle et al., 2000). Based on the versatility of these two models and our extensive experience in utilizing them for both acute and long‐term studies, we will in the following sections discuss their relevance for the study of PD‐related features.

## 4 THE THY1‐HαSYN (L61) MOUSE MODEL

The L61 tg mice were developed on a C57BL6/DBA2 background in the laboratory of Eliezer Masliah (Rockenstein et al., 2002). The model overexpresses (∼ten‐fold increase in expression) human WT *SNCA* under the Thy‐1 promoter. The L61 tg mice have been shown to reproduce several features of PD (Rockenstein et al., 2002), including robust α‐syn brain pathology, alterations in dopamine homeostasis, and motor impairments (Lam et al., 2011) (Figure 2). In these mice, insoluble α‐syn inclusions containing high levels of phosphorylated α‐syn are present in dopaminergic neurons in substantia nigra. Moreover, L61 tg mice develop a progressive loss of striatal dopamine from 14 months of age, which is preceded by an increase in extracellular dopamine release that may explain why these mice have a hyperactive phenotype (Chesselet et al., 2012). In a recent study in these mice, we described an age progressive increase in the levels of oligomeric α‐syn that recapitulates the age progressive nature of PD (Roshanbin et al., 2021). Moreover, we reported a region‐specific diversity in the distribution of phosphorylated (p129) α‐syn. Aggregated α‐syn was observed in the perikaryons of cortical and hippocampal neurons, whereas a more diffuse presence of synaptic α‐syn was detected in brain stem neurons (Roshanbin et al., 2021). Since the Thy1‐hαSyn transgene is X‐linked, male mice develop a more severe phenotype. Other mouse models expressing human WT *SNCA* with different promoters, such as the PDGFβ promoter (Masliah et al., 2000), develop neuropathology by 2 months of age, resulting in motor dysfunction and nerve terminal degeneration in the basal ganglia. Similarly, overexpression of mutated (A53T) *SNCA* under the Thy‐1 promoter creates a robust phenotype with intra‐ and extracellular α‐syn deposits leading to neurodegeneration and severe motor dysfunctions, including spasticity and tremors (Martin et al., 2014). By instead using the *CaMKIIα‐tTA* promoter, degeneration of neurons in the SNpc and hippocampus as well as reduced hippocampal neurogenesis, leading to progressive motor decline and cognitive impairment, have been demonstrated (Nuber et al., 2008). Moreover, conditonal postnatal overexpression of SNCA, also using the *CaMKIIα‐tTA* promoter, was found to result in an isolated hippocampal degeneration (Disease, Injury, & Prevalence, 2016).

## 5 THE (THY‐1)‐H[A30P]α‐SYN (A30P) tg MOUSE MODEL

The A30P tg mice show a roughly two‐fold overexpression of *SNCA*, elevated α‐syn oligomer levels, and develop severe age‐dependent motor impairments (Kahle et al., 2000). As previously shown by us, these mice display high levels of α‐syn oligomers/protofibrils in the spinal cord after 12 months of age (Lindstrom et al., 2014). Further, the levels of such α‐syn species correlate with motor symptoms (Figure 2). In line with previous findings of a limited impact of α‐syn pathology on the dopaminergic system in A30P tg mice (Neumann et al., 2002), we found a minor loss of tyrosine hydroxylase (TH) in the midbrain (Ekmark‐Lewen et al., 2018).

Expressing the A30P mutation by the mouse prion promoter was shown to result in α‐syn accumulation in midbrain dopaminergic cells and dysregulation in the dynamics of presynaptic dopamine release, while the overall number of dopaminergic neurons and levels of striatal dopamine remained unchanged. These alterations in dopamine homeostasis were correlated with behavioral phenotype, hypolocomotion, and motor impairments (Yavich et al., 2005). When instead expressing the double mutation A30P/A53T with the Rat TH promoter, altered dopaminergic terminals and an age‐related decline in motor coordination could be demonstrated (Richfield et al., 2002).

## 6 PD SYMPTOMS AND HOW TO ASSESS THEM IN MICE

Parkinson's disease is diagnosed based on the presence of bradykinesia together with muscular rigidity and/or resting tremor and impaired gait (Poewe et al., 2017). Motor symptoms typically develop in late stages of the disease, when nearly 60% of the dopaminergic neurons in SNpc are either affected or lost (Savitt et al., 2006). The nonmotor symptoms of PD include cognitive impairment, sleep disturbances, anxiety and depression, as well as gastrointestinal and sensory abnormalities linked to autonomic nervous system dysfunction (Kalia & Lang, 2015).

**FIGURE 3:**
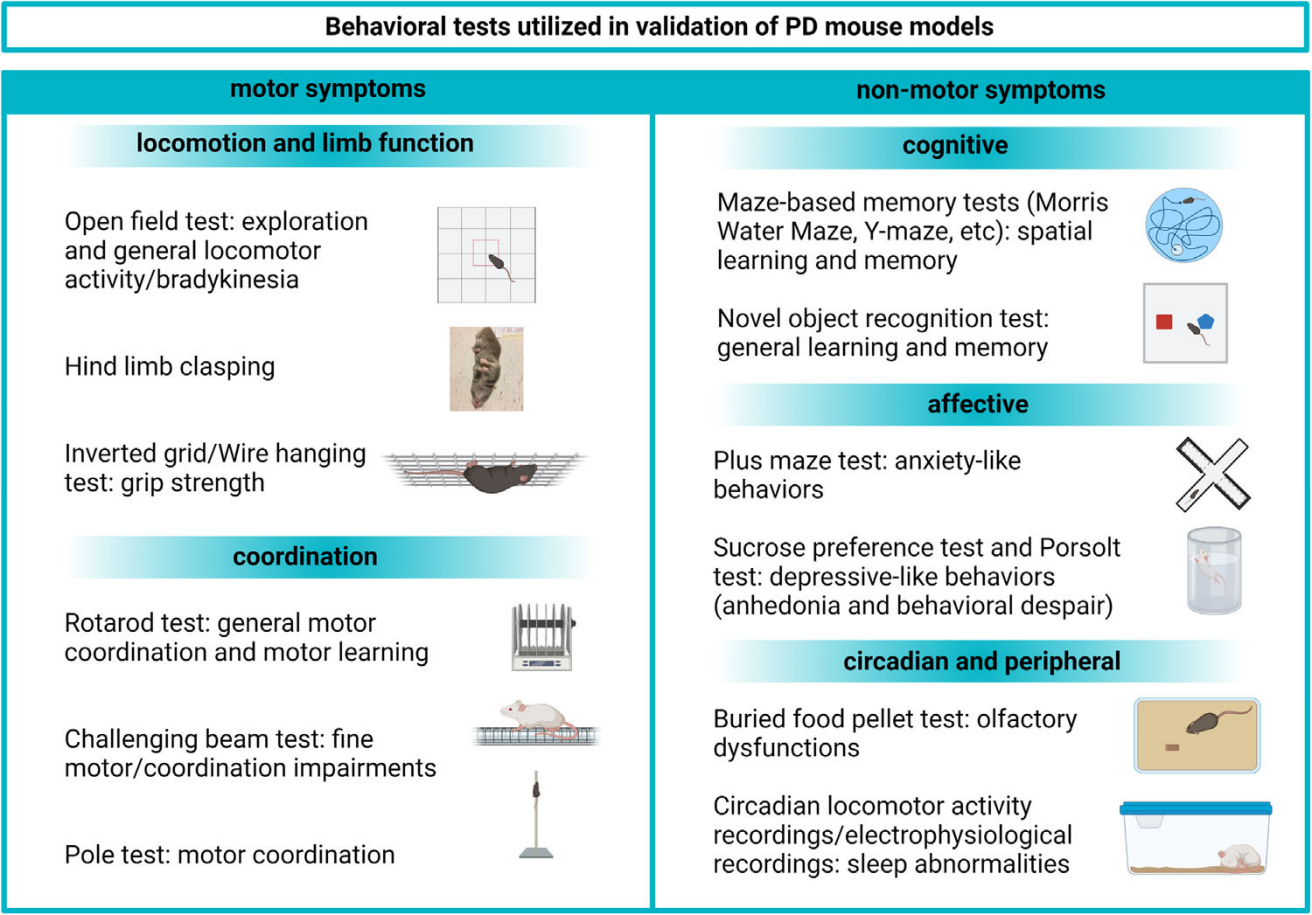
Summary of behavioral tests and procedures used to assess the most common motor and nonmotor symptoms observed in mouse models of PD. Created with BioRender

Ideally, mouse models of PD should recapitulate both the motor and nonmotor disease‐related symptoms. Considering differences in behavioral manifestations of neurodegeneration between mice and humans and the complexity in the clinical picture of PD, it is challenging to generate models that truly mirror the disease. We will here describe some of the advantages and limitations in translating PD symptoms into behavioral features that can be registered and quantified in mouse models. The most commonly used behavioral tests used for the validation of PD mouse models are presented in Figure 3.

## 7 MOTOR SYMPTOMS

The PD‐related motor symptoms usually appear unilaterally and the side domination in the severity of symptoms often lasts while the disease progresses. The disease onset has a broad age range, from the early 40s to late 80s. Early‐onset PD, with the first symptoms observed in patients already before the age of 40, typically has a monogenic cause.

Currently available animal models of PD allow for assessment of various aspects of motor function, although the motor symptoms in mice might not directly mirror those observed in patients.

## 8 GENERAL HEALTH AND OVERT MOTOR IMPAIRMENTS

Regardless of automatically quantifiable measurements of mouse behavior, we would like to stress that the attention of the investigator should always be directed toward the general health status of the animal. Body weight and temperature as well as abnormal behavior, such as hind limb clasping, gait abnormalities, hunchback posture, or unusual tail movements should be observed and noted. Basic neurological scoring according to a premade criterion can also be easily performed (Fernagut & Chesselet, 2004; Fernagut et al., 2003; Guyenet et al., 2010).

Multiple neurological and general health‐related abnormalities have been reported in aged L61 and A30P tg mice. The L61 mice are characterized by early hind limb clasping and stress‐related behaviors (Roshanbin et al., 2021). Moreover, while in A30P tg mice, the abnormalities steadily progress for several months, in L61 tg mice, the occurrence of severe symptoms usually leads to quick worsening of their wellbeing to the point where they have to be euthanized. Also, male L61 tg mice have a reduced life span compared to both female L61 tg mice and age‐matched controls (Roshanbin et al., 2021). The A30P tg mice show progressive fine motor impairments (Ekmark‐Lewen et al., 2018) as well as impaired locomotor performance already during their first year (Neumann et al., 2002) as well as later in life (Freichel et al., 2007). Furthermore, unsteady gait, progressive weakening of the extremities as well as abnormal postures and movements of the tail can usually be observed in aging A30P animals. Consistent with the progressive phenotype, at the end stage of life, animals display a hunchback posture and spastic paralysis of the hind limbs and are unable to feed themselves (Neumann et al., 2002).

## 9 LOCOMOTION

Bradykinesia can be assessed through various types of locomotor function scoring. The open field test (reviewed in [Kraeuter et al., 2019]) is currently the most commonly used method for this purpose. A typical assessment of open field activity includes analyses of locomotion (velocity, distance traveled), signs of anxiety, and stereotypical behaviors (Figure 3). In the context of neurodegeneration, a decrease as well as an increase in open field locomotion can reflect abnormalities in brain function. In PD mouse models, hyperactivity is typically attributed to aberrant dopamine levels in the brain (Lam et al., 2011). Aside from neurodegeneration, a decreased activity in the open field test might also be interpreted as a sign of stress, especially when it is accompanied by an increase in stereotypical behavior. Another important parameter is thigmotaxis, defined as a tendency to stay close to the walls and to move around in the periphery of the arena rather than in its center. This type of behavior is a typical indicator of anxiety‐like behavior (Kraeuter et al., 2019).

Both L61 and A30P tg mice display various abnormalities in locomotion and activity in the open field test. In 7‐months‐old L61 tg mice, an increase in the time spent in motion, total distance traveled in the arena, and movement speed were linked to elevated extracellular dopamine levels in the striatum (Lam et al., 2011). In 14‐months‐old L61 tg mice, a decreased locomotion in the open field was observed, which indicates a progression of neuropathology (Chesselet et al., 2012). Moreover, these mice show decreased movement in their home cage and during handling as well as reduced grooming, as indicated by the state of their fur, which is an indirect sign of further impairments in motor skills (Chesselet et al., 2012). Additionally, we showed that L61 tg mice of both sexes display hyperactivity and thigmotaxis in the open field test (Roshanbin et al., 2021).

In A30P tg mice, we could also describe a decrease in locomotor activity in the multivariate concentric square field (MCSF) test (this test is discussed in more detail later) at 8 months of age (Ekmark‐Lewen et al., 2018), while a significant increase in open field activity was shown in 12‐months‐old, but not in 4‐months‐old, animals (Freichel et al., 2007). The discrepancies in these observations might be an effect of the progressive nature of behavioral abnormalities in α‐syn overexpressing mice as well as the low selectivity of a general locomotor activity that is typically assessed via open field testing.

The rotarod test (Figure 3) is commonly used to assess general motor function and motor coordination in rodents and was created to quantify the effects of neurological abnormalities. Typically, the test consists of several trials in which the mice are placed on a rod rotating at a fixed or gradually increasing speed, after which the time to fall from the rod is registered (Brooks & Dunnett, 2009; Sedelis et al., 2001). There are relatively few studies that have assessed rotarod performance in L61 and A30P tg mice. However, in L61 tg mice, a significant dysfunction in comparison to WT littermates as well as an age progressive decrease in the latency to fall was found (Rabl et al., 2017). In comparison to WT controls, young A30P tg mice were not shown to differ in latency to fall although, starting from 12 months of age, a progressive worsening in their ability to stay on the rod was reported and a significant difference between tg and WT mice was observed at the age of 17 months (Freichel et al., 2007).

While both open field and rotarod tests can be fully automated and easy to use, multiple findings suggest that they might not be sensitive enough to detect smaller abnormalities in the dopamine system (Fleming et al., 2013). Some models, like parkin‐deficient mice, develop subtle alterations in the dopamine system that are not severe enough to cause motor impairments detectable with the rotarod test, whereas their performance in the more sensitive challenging beam test (described later) is significantly impaired (Goldberg et al., 2003). Moreover, in exogenous neurotoxin 1‐methyl‐4‐phenyl‐1,2,3,6‐tetrahydropyridine (MPTP) treated mice, significant gait abnormalities and an impaired performance in an inverted grid test were observed, while their rotarod performance remained unaffected (Tillerson et al., 2002).

Taken together, the subtle and often progressive abnormalities induced by α‐syn pathology might not always be detectable using tests that only assess simple parameters of locomotion. Additionally, considering the nonmotor functions of the dopamine system, assessments of mouse behaviors related to anxiety, depression, and reward system might provide further insight into the relationship between pathology and symptoms. Therefore, implementing a battery of tests that include examination of fine motor impairments as well as other features might be more effective in unraveling the full spectrum of abnormalities that can be attributed to α‐syn deposition in the brain.

## 10 CLASPING BEHAVIOR, GAIT DEFICIENCIES, AND FINE MOTOR IMPAIRMENTS

### 10.1 Clasping behavior

Limb clasping (Figure 3) is a feature that can be a relevant mouse analogue of the pathological grasp reflex observed in PD patients with stages 3 and 4 of the Hoehn and Yahr scale (Huber & Paulson, 1986; Lalonde & Strazielle, 2011). Healthy adult mice extend all four paws while picked up by the tail over a flat surface to ensure a safe position when reaching said surface is possible. Abnormal flexion response induced by spinal cord resulting in paw‐clasping can be observed in basal ganglia dysfunction and is exacerbated by MPTP dopamine depletion (Lalonde & Strazielle, 2011). We recently showed age progressive hind limb clasping in both male and female L61 tg mice, whereas no clasping could be seen in age‐ and sex‐matched WT controls (Roshanbin et al., 2021).

### 10.2 Motor coordination

The pole test (Figure 3), which measures motor coordination, is a well‐known tool to assess motor abnormalities resulting from dopamine depletion (Matsuura et al., 1997). Therefore, it is being applied for the evaluation of transgenic (Kelm‐Nelson et al., 2018; Rousseaux et al., 2012) PD mouse models. In this test, the mouse is placed with its head up on a 50 cm long and 1 cm wide pole, after which the latency to turn and descend from the pole is measured. This test has been adopted in several trials on *SNCA* transgenic mice (Chesselet et al., 2012; Matsuura et al., 1997). In the L61 tg mice, a robust and age progressive impairment in pole test performance (decreased latency to turn and descend time) was reported already at 2 months of age (Chesselet et al., 2012; Fleming et al., 2004).

### 10.3 Grip strength

Grip strength is typically measured in two ways: On the inverted grid, where the mouse is required to cling onto an inverted surface (usually a wired grid or a home cage cover) or on a wire (where mice are required to cling onto a hanging wire using their forepaws) (Figure 3). The mouse is kept on a grid/wire for a given time or until fall and the time spent on the grid/wire is registered (Brooks & Dunnett, 2009; Tillerson et al., 2002). These types of tests are used in drug discovery or in other forms of PD research and can indicate progression of motor abnormalities over time. Grip strength dysfunction can be correlated with a loss of dopaminergic markers in the striatum and is reversible after l‐DOPA treatment (Tillerson & Miller, 2003; Tillerson et al., 2002). In L61 tg mice, a significant decrease in step distance was observed already at 3 months of age (Fleming et al., 2004).

### 10.4 Gait analysis and stride length

Reduced stride length is one of the features that characterizes parkinsonian gait impairments. The analysis of gait during normal walking can provide important information regarding motor coordination and synchrony of movement, which translates well from animal to human studies. In mice the footprint test is used, in which front and hind paws are painted with a nontoxic paint of different colors to allow for a distinct footprint on an absorbing paper while the mouse is walking through a narrow space. The measurements include stride length and width, the distances between paw prints, overlap between front and hind paws, range of stride length, difference between the longest and the shortest stride, as well as other parameters (Brooks & Dunnett, 2009). In rodents, a reduced stride length is considered to be an indication of basal ganglia dysfunction. Pharmacological depletion of dopamine or impairments in dopamine signaling as well as striatal lesions and nigral cell loss are known to lead to significant impairments in stride length and the severity of such impairments can be directly attributed to the magnitude of dopamine deficiency (Fernagut et al., 2002). Therefore, the assessment of stride length is considered to be a valuable addition to the evaluation of PD mouse models. Regardless, in 8‐months‐old L61 tg mice, no significant abnormalities were observed, although there was a trend toward more variable stride lengths and shorter steps (Fleming et al., 2004).

### 10.5 Progressive motor and coordination impairments

Considering that the motor impairments observed in *SNCA* overexpressing mice might be subtle, tests evaluating fine motor impairments are being implemented as a complement to tests that can detect overall and robust deficiencies. The challenging beam traversal test is performed on a beam of decreasing width, covered with a metal grid (Figure 3). The traverse time, number of steps, number of errors (not placing a paw correctly onto the grid), and the error/step ratio are typically scored. The test allows for the assessment of the animal's control of fine movement and coordination, which are both required to accurately grasp the grid. Moreover, it has been suggested that this test is less influenced by the animal body weight than the rotarod and pole tests, which should be particularly beneficial when studying aged rodents (Fleming et al., 2013). The challenging beam traversal test is widely used in long‐term studies, due to its effectiveness in detecting age/time progressive changes in motor function (Ekmark‐Lewen et al., 2018). Although it can be used repeatedly, in studies involving neurodegeneration, excessive repeats should be used with caution, due to potential benefits of training in retaining motor skills that would otherwise be worsened. The L61 tg mice show age progressive deficits in challenging motor tests, which are especially profound in males while females show a less robust phenotype (Chesselet et al., 2012). In a recent study, we showed that A30P tg mice display significant fine motor impairments that can be detected already at 2 months of age, with a progressive deterioration up to 11 months of age (Ekmark‐Lewen et al., 2018).

## 11 NONMOTOR SYMPTOMS

Nonmotor features in PD patients can be classified as neuropsychiatric abnormalities (anxiety, depression, executive function impairments, visuospatial deficits, psychosis, apathy, aggressiveness, disinhibition and cognitive impairment/dementia), circadian rhythm abnormalities (impaired REM sleep behavior, insomnia and daytime sleepiness, restless leg syndrome), and gastrointestinal abnormalities (dysphagia, constipation, swallowing difficulties, hypersalivation) (reviewed in [Jellinger, 2015]). Some of these symptoms are believed to be related to α‐syn pathology in the CNS and/or in the nervous system of the gastrointestinal tract. Unfortunately, not all of these features can be reproduced in mouse models, due to either the character of α‐syn pathology induced in the mice or to species‐related differences in the behavioral repertoire. Nevertheless, some of the symptoms can be fairly well modeled and in this section we will discuss how the most important nonmotor impairments of prodromal and late‐stage PD can be investigated in *SNCA* tg mice and what value this may have for the evaluation of novel treatment strategies.

### 11.1 Cognitive dysfunctions

About 80% of PD patients develop dementia and 20–55% of nondemented PD patients display mild cognitive impairment (reviewed in [Goldman & Litvan, 2011]). The occurrence of dementia is usually preceded by a longer period of progressive cognitive impairment, although a large variation between patients can be observed (reviewed in [Watson & Leverenz, 2010]). There are several types of cognitive impairments, which are probably related to the appearance of α‐syn pathology in brain areas linked to specific cognitive functions. Cognitive impairments, with or without dementia, are often debilitating for the patients and provide an additional burden for caregivers. Therefore, measures of cognitive function should be an integral part of the evaluation of new PD therapies.

### 11.2 Learning and memory

Memory processes engage multiple brain areas and α‐syn pathology can affect different aspects of memory formation and retention. Contrary to declarative and episodic memory (which are mainly dependent on hippocampus, neocortex, and other higher order brain structures), procedural memory (which is related to the basal ganglia, motor cortex, and cerebellum) can be particularly affected by PD‐like pathology.

In PD patients, memory impairments can be diagnosed using verbal or nonverbal memory tests that measure immediate recall, delayed recall, and recognition. Multiple studies have reported that immediate and delayed learning of particular stimuli sequences can be impaired in PD (Aarsland et al., 2009; Goldman et al., 2018; Muslimovic et al., 2007).

In humans, cognitive abilities are most commonly assessed with tests that involve verbal communication and/or abstract thinking, whereas cognition and memory in rodents need to be assessed with consideration of the ethological relevance of their behaviors. There are multiple ways of testing memory in mice, e.g., with visuospatial memory tests (Morris water maze [MWM] and other maze‐based tests) and recognition memory tests (e.g., novel object recognition [NOR]) (Figure 3).

Mouse models overexpressing WT or mutated human *SNCA* show various cognitive impairments (reviewed in [Hatami & Chesselet, 2015]). In comparison with age‐matched wild‐type littermates, the L61 tg male mice show a decreased number of spontaneous alternations in the Y‐maze and abnormalities in NOR and object–place recognition tests. Additionally, alterations in reversal learning can be observed, indicating a reduced cognitive flexibility. Cognitive abnormalities in this mouse model can be detected at an early age, preceding the onset of motor impairments, which correlates with an increase of extracellular dopamine levels in the striatum (Magen et al., 2012). The A30P tg mice show cognitive abnormalities later in life. A significant impairment in fear conditioning was observed at 16 to 19 months of age, and these impairments were correlated with an age‐dependent increase of cellular p129 α‐syn pathology in the cortex and with the development of synaptic p129 pathology in the brain stem and fear conditioning‐related areas, including amygdala (Schell et al., 2012, 2009). Impairments in the MWM test were also observed in aging but not in young, A30P tg mice. The A30P tg mice performed worse than WT in the second probe trial, 24 h after last acquisition probe, indicating abnormalities in long‐term memory (Freichel et al., 2007).

As another particularly relevant aspect, long‐term motor function assessment should be included in the evaluation of PD mouse models. In prolonged treatment studies, in which animals are repeatedly subjected to tests assessing motor impairments at designated time points, the often neglected learning factor can potentially affect the interpretation of results and the estimation of treatment effects. Whereas some authors control for learning effects in their experimental designs (Magen et al., 2012), other investigators do not state if any such analyses were performed.

### 11.3 Anxiety and depression in PD

Anxiety and depression affect approximately 40% of patients with PD and the frequency of depression is more common than for other similarly debilitating conditions (Marsh, 2013; Remy et al., 2005). As a possible neuroanatomical correlate to this observation, PD brains have been found to display a reduced gray matter density (Blonder & Slevin, 2011; Feldmann et al., 2008) as well as Lewy body pathology in areas connected with mood‐related serotonergic pathways (Parkkinen et al., 2008; Tan et al., 2011). Moreover, the abnormalities observed in adrenergic and serotonergic innervation may be attributable to the anxiety in PD (Prediger et al., 2012).

There are multiple ways of testing anxiety in mouse models (reviewed in [Lezak et al., 2017]). Anxiety and fear in both rodents and humans can be reflected by similar behavioral features, including freezing, increased vigilance, and/or decreased locomotor activity. However, rodent behaviors cannot be directly translated to reflect the human trait of anxiety, which can be conceptualized psychologically and measured using questionnaires. Therefore, behavioral studies performed on mice utilize the term anxiety‐like behavior. Typical models of anxiety‐like behavior are based on creating an ethologically relevant environment to assess approach versus avoidance, vigilance, or defensive behaviors. Latency to approach or time spent in a potentially dangerous environment or exploring a novel object is used as an indirect indicator of anxiety. Several aspects of anxiety‐like behaviors, including exploratory behaviors, rearing, thigmotaxis, or hypoactivity/freezing, can also be quantified in the open field test (Bailey & Crawley, 2009).

Modeling complex and multidimensional disorders, such as depression, in rodents is highly challenging. Tests designed to assess behavior that resembles depressive states in humans typically utilize two features of mouse behavior: Anhedonia, conceptualized as a lack or a diminished preference for reward and a lack of defensive response in threatening conditions (so called “behavioral despair”) (Porsolt et al., 1977). Due to the limited validity of these procedures, their accuracy and relevance remain a topic of discussion (Krishnan & Nestler, 2011; Planchez et al., 2019; Wang et al., 2017).

The forced swimming and elevated‐plus maze tests (Figure 3) are the most frequently used methods to assess depressive‐ and anxiety‐like behaviors in mice. Importantly, performance in both of those tests can also be significantly affected by motor impairments. Therefore, when including results of these tests as part of the validation process, it is important to consider the influence of motor impairment severity in the overall assessment.

Both the L61 and A30P tg mice feature widespread α‐syn brain pathology, including pathology in areas that can be connected with mood disorders (Rockenstein et al., 2002). The L61 tg mice display more anxiety‐like behaviors than their WT littermates. At 4 months of age, L61 tg mice were shown to have an increased freezing duration in response to a conditioned stimulus, but differences in fear extinction between L61 tg and WT mice could not be observed (Torres, et al., 2010). Interestingly, the hyperactivity of L61 tg mice can be a factor that masks their anxiety phenotype (Lam et al., 2011; Mulligan et al., 2008). On the contrary, we showed that A30P tg mice display impairments in general locomotion in the MCSF test, but that they do not seem to display typical indicators of significant impairments in exploratory behaviors (Ekmark‐Lewen et al., 2018). We are currently lacking assessments of depression‐like behaviors in *SNCA* tg mouse models, as the presence of motor symptoms complicates the observations of such features. Utilization of tests like forced swimming could be viewed as ethically questionable, especially at later stages of α‐syn pathology when motor symptoms might limit the animals’ mobility and thereby affect the test outcome.

Taken together, the data from previous studies suggest that assessments of anxiety‐like behaviors might be a valuable addition to PD mouse model evaluation, although the procedures need to be selected with caution and understanding of the potential influence of fine motor impairments on the test outcomes.

### 11.4 Olfactory and gastrointestinal dysfunctions

Olfactory dysfunction is a frequent preclinical feature of PD (Liepelt‐Scarfone et al., 2011). The occurrence is most likely an effect of α‐syn pathology in the olfactory bulb and upstream olfactory nuclei (Attems et al., 2014; Duda, 2010; Silveira‐Moriyama et al., 2009), whereas the olfactory epithelium usually remains unaffected (Witt et al., 2009). These findings indicate that olfactory dysfunction in PD occurs due to impairments in the central olfactory pathways, rather than in the peripheral sensory nerve fibers (Dickson et al., 2009). Similar to olfactory deficits, gastrointestinal dysfunction can occur in PD patients as early as 20 years before the development of motor symptoms (Poewe, 2008; Savica et al., 2009). Gastrointestinal dysfunction is likely caused by α‐syn pathology in the autonomic nervous system and in brain areas controlling autonomic nervous system input, including the dorsal motor nucleus of vagus, enteric nervous system, adrenal medulla, and sympathetic ganglia (Boeve et al., 2007; Braak et al., 2006; Gries et al., 2021; Jellinger, 2015).

In mice, lack of preference for sniffing other animals’ scent or prolonged latency to find a hidden food pellet (Figure 3) after a period of food restriction are considered as measures of olfactory dysfunction. Gastrointestinal dysfunction can be indicated with a delayed basal colonic transit and increased fecal pellet output in response to stress.

Data suggest that L61 tg mice develop both olfactory and gastrointestinal dysfunctions (Fleming et al., 2008). From an early age, such mice show reduced sniffing time of other animals’ scent. Additionally, L61 tg mice show abnormalities in olfactory discrimination and habituation time to new scents and detection (increased latency to finding buried food pellet), although the deficits seem to lack the progressive character observed in PD and a complete loss of olfaction cannot be observed (Fleming et al., 2008). While there is so far no evidence connecting α‐syn pathology with olfactory dysfunctions in PD mouse models, studies performed on A30P tg mice suggest that the A30P mutation affects the integration and survival of neurons that have newly migrated to the olfactory bulb (Neuner et al., 2014).

The data on gastrointestinal dysfunction in tg mouse models overexpressing *SNCA* are limited, but studies suggest that these preclinical PD symptoms can be attributed to α‐syn pathology (Greene, 2011). Reduced basal colonic transit and increased fecal pellet output in response to stress were observed in 12‐months‐old L61 tg mice (Wang et al., 2008), whereas younger animals showed a reduction in fecal pellet output after stress (Chesselet et al., 2012).

### 11.5 Circadian rhythm and sleep dysfunctions

Sleep disturbances are considered to be one of the main preclinical symptoms of PD. The first abnormalities appear long before the first manifestations of motor symptoms and include insomnia as well as corresponding daytime sleepiness, sleep fragmentation, and REM sleep behavior disorder (RBD) (Claassen et al., 2010; Iranzo et al., 2006). Up to 50 % of the patients suffering from RBD develop PD symptoms (Boeve, 2010; Boeve et al., 2007; Kim et al., 2018) and the combination of RBD and olfactory impairments is considered to be a sign of manifest α‐syn pathology (Stiasny‐Kolster et al., 2005). The RBD is defined as a loss of REM sleep atonia combined with the manifestations of complex, vigorous behaviors that can be attributed to abnormal suppression of motor activity during REM sleep phases (Bassetti & Bargiotas, 2018). Although animal models of RBD were initially created by pontine tegmental lesions, there is evidence of naturally occurring RBD‐like sleep dysfunctions in animals (reviewed in [Schenck & Mahowald, 2002]). Sleep disturbances or circadian rhythm dysfunction are included in the validation of multiple models of α‐synucleinopathies (Kudo et al., 2011; Shen et al., 2020; Stylianou et al., 2020).

The most common approach to assess circadian rhythm in rodents is to continuously observe locomotor activity of the animal throughout the light/dark cycle (Figure 3). Additionally, recording of animals during sleep allows for a manual scoring of the duration of the different sleep phases, based on behavioral measures, such as locomotion or grooming (Campbell & Tobler, 1984; Kudo, Schroeder, et al., 2011). Disruption of circadian rhythm can also be assessed through electrophysiological recordings in relevant brain areas, especially the suprachiasmatic nucleus (SCN) (Ruiz‐Mejias et al., 2011; Sharma et al., 2010; Wolansky et al., 2006).

The L61 tg mice display age progressive abnormalities in circadian regulation of their motor activity prior to developing PD‐like motor symptoms (Chesselet et al., 2012). The circadian pattern of motor activity, including lower nighttime motor activity and increased fragmentation of running wheel activity, was disrupted already at 3 months of age, a feature that worsened up to 12 months of age. Moreover, L61 tg mice show alterations in temporal distribution of sleep, with an increased sleeping time in late day and a decreased time in late night as well as a reduction in neural activity of the SCN (Kudo, Loh, et al., 2011). In A30P tg mice, alterations in sleep‐related oscillations were shown in medial prefrontal cortex and hippocampus in 2‐ to 3‐months‐old animals, i.e., several months prior to the occurrence of motor symptoms (Stylianou et al., 2020).

## 12 VALIDITY OF ANIMAL MODELS

The validity of animal models is discussed in terms of face‐, construct‐ and predictive validity. Although no animal model is likely to simultaneously fulfill all validity criteria, each model has its advantages and disadvantages. Depending on the priorities of a particular study, these criteria are of different significance. Face validity is considered as the phenomenological and symptomatological similarity between the features of the animal model and the human disease that is modeled, e.g. the degree of descriptive similarity between the behavioral abnormalities or pathological alterations seen in the model system and the human condition. From a symptomatological point of view, the face validity of PD animal models is compromised as the quantifiable mouse behavior outcomes cannot fully recapitulate the complex alterations in the patient behavior.

Nevertheless, both the A30P tg and L61 tg models display a relatively high face validity with respect to the combination of symptoms and pathology, showing similar features in brain as in PD (Kahle et al., 2000; Rockenstein et al., 2002). Moreover, the mice develop a progressive behavioral phenotype with fine motor impairments and memory deficiency (Ekmark‐Lewen et al., 2018; Freichel et al., 2007; Rockenstein et al., 2002). We have recently shown that L61 tg mice have an age‐related increase in brain of α‐syn oligomers and that males, in addition to a more pronounced hyperactivity and thigmotaxis in the open field test, feature increased α‐syn oligomer brain levels from an earlier age, compared to females (Roshanbin et al., 2021). Additionally, most likely due to their more severe α‐syn pathology, males display a higher mortality, which makes them more suitable to acute treatment studies. On the other hand, female mice with a less acute phenotype and longer survival might be more suitable for long‐term treatment studies. (Roshanbin et al., 2021). As for the A30P tg model, no sex‐related differences in α‐syn pathology have been reported.

Construct validity is considered as the degree of similarity between the mechanisms underlying a condition. Multiplications of and mutations in the *SNCA* gene are causative factors for rare familial forms of PD. Therefore, overexpressing human WT or mutated *SNCA*, leading to accumulation of aggregated α‐syn and a progressive PD‐like phenotype, provide a direct link between the disease and the mouse model.

At last, predictive validity describes how well the model allows for extrapolation from one species to another, including humans, and from one condition to another. A model with a high predictive validity shows a good treatment effect using the same drug as for the human condition, i.e., if a treatment is effective in the model it is more likely to be successful also in the patients. The L61 tg model develops moderate striatal dopamine loss and some positive l‐DOPA response (reviewed in [Chesselet & Richter, 2011; Lam et al., 2011]). However, the dopaminergic dysfunction in A30P tg mice is limited and no treatment effect with l‐dopa has been shown, indicating a lower predictive validity with respect to l‐dopa treatment. However, mouse models where significant dopamine loss is not observed still play an important role in studying α‐syn aggregation and evaluation of treatments, including immunotherapy targeting α‐syn oligomers/protofibrils.

## 13 FACTORS AFFECTING BEHAVIORAL OUTCOMES

Analyses of behavioral phenotypes in PD mouse models are often inconsistent as a result of the variability between models. For transgenic α‐syn mice, the various models show different behavioral phenotypes depending on which promoter that is used and which form of human α‐syn that is expressed. Models with a more robust overexpression of α‐syn, such as the L61 tg mice (Roshanbin et al., 2021), show a stronger and more rapidly progressing phenotype than mice with a less pronounced pathology, such as the A30P tg mice (Ekmark‐Lewen et al., 2018). Generally, the severity of PD‐like motor impairments in those models can be correlated with the observed alterations in dopaminergic system (Chesselet et al., 2012; Yavich et al., 2005).

The fact that behavioral studies are subject to the influence of multiple genetic factors highlights the importance of choosing an appropriate background strain (Sultana et al., 2019). Also, environmental factors, such as conditions of housing and testing, animal handling and access to an enriched environment, will have an important impact on the study results (Bailoo et al., 2018; Robinson et al., 2018; Sensini et al., 2020). Another factor that can influence the outcome of behavioral tests, especially those that are more complex, is the coping style. Mice avoid predators and stay alive in their natural environment by using different survival strategies. In general, mice are neophobic, exploratory but risk assessing as well as nocturnal and communicate mainly by scent. However, mice have diverse coping responses, controlled partly by habitual traits and partly by the social environment, particularly the nature of the stressful environment (Carver and Connor‐Smith, 2010). In psychology, coping involves a conscious effort to solve problems to resolve, minimize, or tolerate stress and conflict. The effectiveness of the coping efforts depends on the type of stress and/or conflict, the particular individual as well as the circumstances. Many different coping strategies have been identified also in animals and these features can affect mice performance in behavioral tests and contribute to within‐group variability.

Considering the complexity and variety of factors intertwined in the regulation of mouse behavioral phenotype, it is crucial that validation of novel PD mouse models include a thorough and broad behavioral examination (Brown & Bolivar, 2018).

## 14 ETHOEXPERIMENTAL AND MULTIVARIATE APPROACHES

The ethoexperimental approach to studies of rodent behavior was introduced in the 1980s (Blanchard & Blanchard, 1988). In behavioral studies, there has been a long tradition of using a methodological approach based on ethological perspectives, meaning that efforts should be taken to use native forms of behavior and stimuli that can be derived from the natural environment of the species studied (Blanchard & Blanchard, 2003; Calatayud et al., 2004; Tecott & Nestler, 2004). The ethological tradition emphasizes studies of behavior in natural settings, or in laboratory settings specifically designed to include whatever natural features that are necessary to support a fully developed action pattern.

The need for tests that deal with multiple measures, thus providing a behavioral profile rather than single parameters, has been emphasized (Blanchard et al., 1993; Meyerson et al., 2006). To meet this end, ethoexperimental composite tests have been designed (Blanchard & Blanchard, 1989; Blanchard et al., 2003, 1993; Meyerson et al., 2006).

**FIGURE 4:**
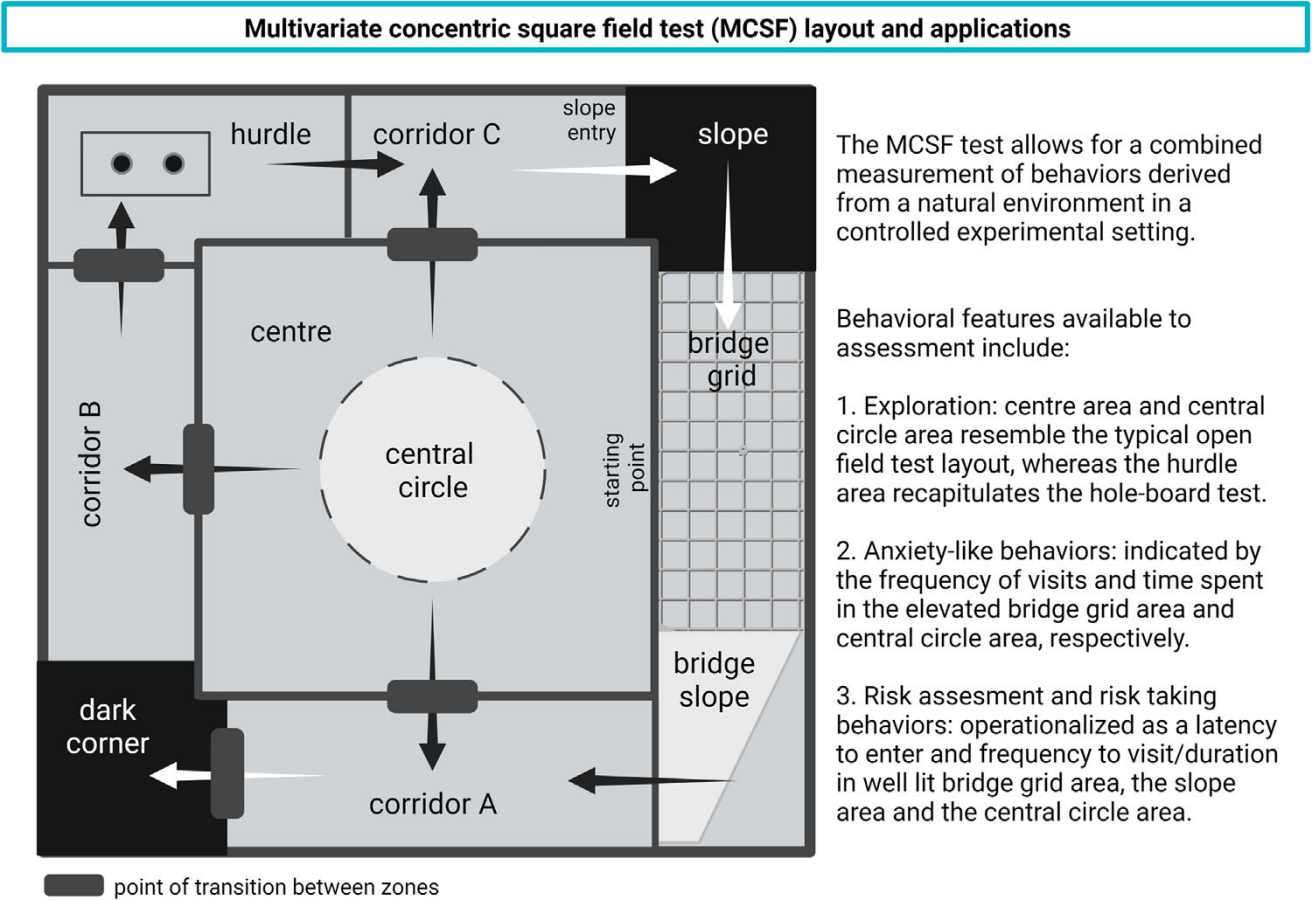
The multivariate concentric square field (MCSF) test is used to assess ethologically relevant behaviors in an environment that recapitulates features observed in the natural habitats of rodents. The arena includes central areas suitable for measurements of basic locomotor activity and exploration, as well as side areas designed to assess specific behavioral features, e.g. anxiety‐like behaviors or risk taking. Created with BioRender

The MCSF test (Figure 4), in which the test subject has a free choice of different environments contained in the same apparatus and session, was first used to identify exploratory strategies in rats (Meyerson et al., 2006). The MCSF test is not coupled to the ambition of measuring a previously specified mental state or function (Meyerson et al., 2006) and the design is based on “forced” exploration, i.e., the animal is released directly into the arena (Belzung, 1999). As opposed to free exploration, when the animal voluntarily enters a novel arena from a familiar home base (start box), this procedure involves inescapable novelty, which may interact with exploratory motivation and risk assessment behavior.

Over the years, the MCSF test has been further developed and used for behavioral profiling in a rat model of alcohol addiction (Karlsson & Roman, 2016; Roman & Colombo, 2009; Roman et al., 2007, 2012), studies of rat adolescent exploratory strategies (Lundberg et al., 2019), outcome studies of experimental traumatic brain injury in mice (Ekmark‐Lewen et al., 2013, 2010, 2016) as well as behavioral profiling of the A30P tg mouse model (Ekmark‐Lewen et al., 2018). In the MCSF test, A30P tg mice display a decreased general activity, an increased rearing, and an increased risk‐taking behavior, as shown by longer visits to the more exposed bridge area compared to controls (Ekmark‐Lewen et al., 2018). The multivariate set‐up provides opportunities for studies of complex behavioral changes that can be measured during one trial, or in a repeated testing design to measure memory effects.

We believe that by combining ethoexperimental and multivariate approaches, researchers have new possibilities for improving behavioral assessments and get better outcome measures in mouse models of α‐synucleinopathies.

## 15 CONCLUSIONS

Researchers studying behavior in animals often face the problem of large in‐group variations, making it difficult to get significant differences between tested groups. There are several factors that can affect the test results in behavioral studies, such as the sex of the animal, motivation of the animal to perform in the test, coping styles, interactions between tests and, not least, how the researcher handles the test procedure.

*So how can we reduce variability?* To measure a general change in perceptive, cognitive, or locomotor functions, a battery of tests needs to be used. In that case, it is important to take into account the “carry‐over effect” between different tests, meaning that the performance in one test can be affected by the previous test. Another aspect to consider is that the least stressful test should be performed first and tests that may cause stress response in the mice should be performed last; otherwise, the stress effect can be carried over to the next test session. To overcome this problem, a multivariate test that measures many different behaviors at the same time can be the solution. Handling of the mice is another important factor that can reduce stress and decrease variability.

*Can variability be something positive?* When comparing studies of new drugs in humans and animals, the treatment effect can often differ, with less marked differences in the clinical trials. These differences can be explained by many factors but clinical trials are not performed in a controlled environment and patients have different genetic backgrounds and sexes, which can explain some of the discrepancies. To be able to correctly assess symptom effects in preclinical drug development it is important to consider these differences and use animals with different genetic backgrounds and of both sexes. A drug that shows a good effect in several mouse models of a disease has a much higher potential to become a drug that can translate into the clinic. In the case of PD, it can therefore be of value to use mouse models that mimic different aspects of the disease.

Importantly, not all PD symptoms can be modeled in mice. However, by using an ethoexperimental approach in the design of behavioral experiments, the clinical relevance can increase and lead to better test results with less variability.

Taken together, the α‐syn overexpressing mouse lines used in PD research and drug testing develop several symptoms related to the human condition. In combination with relevant and sensitive behavioral tests, these models provide an appropriate experimental platform for studies of symptoms related to PD and other α‐synucleinopathies.

## CONFLICT OF INTEREST

Martin Ingelsson is a paid consultant to BioArctic AB. The other authors do not have any conflict of interest.

### PEER REVIEW

The peer review history for this article is available at https://publons.com/publon/10.1002/brb3.2628.

## Data Availability

Data sharing is not applicable to this article as no new data were created or analyzed in this study.
